# Huntington’s disease iPSC models—using human patient cells to understand the pathology caused by expanded CAG repeats

**DOI:** 10.12703/r/11-16

**Published:** 2022-06-28

**Authors:** Julia Kaye, Terry Reisine, Steven Finkbeiner

**Affiliations:** 1 Center for Systems and Therapeutics, Gladstone Institutes, San Francisco, CA, USA; 2 Independent Scientific Consultant, Santa Cruz, CA, USA; 3 Taube/Koret Center for Neurodegenerative Disease Research, Gladstone Institutes, San Francisco, CA, USA; 4 Department of Neurology and Physiology, University of California, San Francisco, CA, USA

**Keywords:** induced pluripotent stem cells (iPSCs), Huntington’s disease, neurodegeneration, neurodegenerative disease

## Abstract

A major advance in the study of Huntington’s disease (HD) has been the development of human disease models employing induced pluripotent stem cells (iPSCs) derived from patients with HD. Because iPSCs provide an unlimited source of cells and can be obtained from large numbers of HD patients, they are a uniquely valuable tool for investigating disease mechanisms and for discovering potential disease-modifying therapeutics. Here, we summarize some of the important findings in HD pathophysiology that have emerged from studies of patient-derived iPSC lines. Because they retain the genome and actual disease mutations of the patient, they provide a cell source to investigate genetic contributions to the disease. iPSCs provide advantages over other disease models. While iPSC-based technology erases some epigenetic marks, newly developed transdifferentiation methods now let us investigate epigenetic factors that control expression of mutant huntingtin (mHTT). Human HD iPSC lines allow us to investigate how endogenous levels of mHTT affect cell health, in contrast to other models that often rely on overexpressing the protein. iPSCs can be differentiated into neurons and other disease-related cells such as astrocytes from different brain regions to study brain regional differences in the disease process, as well as the cell-cell dependencies involved in HD-associated neurodegeneration. They also serve as a tissue source to investigate factors that impact CAG repeat instability, which is involved in regional differences in neurodegeneration in the HD brain. Human iPSC models can serve as a powerful model system to identify genetic modifiers that may impact disease onset, progression, and symptomatology, providing novel molecular targets for drug discovery.

## Introduction

Huntington’s disease (HD) is caused by the expansion of a CAG repeat in the Huntingtin gene (*HTT*), leading to the expression of a mutant protein (mHTT) with an expanded polyglutamine domain near its N-terminus. Progress towards a cure has been slow, in part because the function of the normal HTT protein is unclear and because the expanded gene and protein appear to impinge on multiple biological processes. The disease manifests as progressive degeneration of striatal neurons and has been modelled in a variety of systems, including animal models and animal or human cell *in vitro* systems.

A major advance for *in vitro* cell modeling of HD was the development of induced pluripotent stem cell (iPSC) technology by groups led by Yamanaka^1^ and Thomson^2^. This technology allows somatic cells to be reprogrammed into a pluripotent state and subsequently differentiated into cell types of interest^1,2^. Unlike other cell models to study HD, human iPSCs retain the genome of their patient source, making them a valuable tool to investigate the genetic contributions to disease. Because iPSCs can be propagated indefinitely, they provide a nearly inexhaustible source of patient-derived material to study disease mechanisms and for use in drug discovery.

The methods to efficiently generate iPSCs have evolved over time and have been extensively reviewed^3,4^. The initial methods used viruses expressing reprogramming factors^1,2,5^. This approach has drawbacks in that the virus would randomly integrate across the genome, thereby causing genomic insults to the DNA that could alter gene expression in unwanted and unknown ways. More advanced methods have been developed that reprogram cells without leaving a genomic fingerprint^4,6^. These include using non-integrating episomes^7^, mRNA^8^, or small molecules^9,10^. Most of the iPSC models with expanded CAG repeats reviewed here were made with non-integrating episomal vectors (Table 1).

**Table 1.** Induced pluripotent stem cell lines to study Huntington’s disease pathogenesis.

iPSC line*	CAG repeat size**	Summary of cellular phenotypes in HD i-tissues compared with controls***
CS97iHD-180nX (GM09197)^18^	180	• ***Gene expression changes:*** ↑*&*↓ *in ECM signaling, synapse assembly, axon guidance,* *neurotrophic signaling, and the BDNF pathway in cortical i-neurons*^31^, *↑ in TGF-β pathway* *and other developmental pathways in i-NSCs*^32^, ↑*&*↓ *in neuronal development pathways,* *cell cycle, proliferation, axon guidance, and nervous system function pathways in* *i-NSCs*^18^ ** Functional phenotypes:** • ↑&↓ in fatty acid metabolism and PPAR signaling in i-NSCs^32^ • ↑ H3K9 methylation in iPSCs^33^ • ↑ ATM and P53 protein levels in iPSCs^34^ • ↑ P53 phosphorylation in iPSCs^34^ • ↑ γH2AX phosphorylation in iPSCs^34^ • ↑ cell death^18^ and ↑ susceptibility to cell death after growth factor withdrawal^32,35^ as well as ↑ susceptibility to cell death after exposure to stressors such as glutamate, 3-MA, H_2_O_2_ in i-neurons^18^ • Proteasome inhibition by MG132 or autophagy inhibition by bafilomycin induces mHTT aggregates in iPSCs^14,36^ • Neuronal patterning and rosette formation^32^ is delayed • OCT4 remains high after neuronal induction in i-NPCs^37^ • ↓ intracellular ATP levels in i-NSCs^18,19^ • ↑ levels of caspase-3/7 activity in i-NSCs^18^ • ↑ number of TUNEL-positive staining iPSCs^18^
CS109iHD-109nX /HD109i.1(ND39258)^18,38^	109	• ***Gene expression changes:*** ↓ *P53 expression in i-NSCs*^39,40^, ↑*&*↓ *in neurodevelopmental* *pathways in i-neurons^17^,* ↑ *in TGF-β pathway i-neurons*^17,38,41^, ↑*&*↓ *in ECM signaling* *in i-neurons^17^ and i-NPCs*^18^, ↑*&*↓ *in synapse assembly, axon guidance, neurotrophic* *signaling, and BDNF pathway in i-neurons*^17^, ↑*&*↓ *in ECM organization, synapse assembly,* *axon guidance, neurotrophic signaling, and the BDNF pathway in cortical i-neurons*^31^, ↑*&*↓ *in neurodevelopmental pathways, DNA damage, and apoptosis in iPSCs*^38^ *and others.* ** Functional phenotypes:** • ↑ nuclear envelope abnormalities in i-NPCs^42^ • Mis-localization of nuclear pore proteins and alterations of nucleocytoplasmic transport in i-neurons^43^ • ↑ susceptibility to cell death after growth factor withdrawal in i-neurons^35^ • ↑&↓ epigenetic alterations in i-neurons^17^ • Alterations in neuronal patterning and rosette formation is delayed and OCT4 remains high after neuronal induction in i-NPCs^32,37^ • Delay of mature electrophysiological currents in i-neurons^31^ • ↑&↓ neurite length in i-neurons^17,31^ • ↓ intracellular ATP levels iPSCs and i-neurons^18,19,44^ • ↓ bioenergetics and ↓ glycolytic capacity in i-neurons and i-NSCs^18,19,44^ • CAG repeat instability in iPSCs and i-NPCs18 over passage as well as over time during differentiation^45^.
CS77iHD-77nX^31^	77	• ***Gene expression changes:*** ↑*&*↓ *in ECM organization, synapse assembly, axon* *guidance, neurotrophic signaling, and the BDNF pathway in cortical i-neurons*^31^. ** Functional phenotypes:** • Delay of mature electrophysiological currents in i-neurons^31^ • ↓ neurite length in cortical i-neurons^31^
HD76^46^		• ↑ SOCE calcium currents i-neurons^46^ • ↑ mHTT protein expression in i-neurons^46^ • ↑ VGCC currents in i-neurons^46^
HD-iPS4^12^, HD and HD2^15^ HD-iPSC(Q71)^12^	72 (Some studies^33^ report this line as having 71 CAG repeats)	• ***Gene expression changes:*** ↑ *in DNA damage pathways*^15^, ↑ *in oxidative stress*^15^, ↓ *in cytoskeletal genes in i-neurons^15^,* ↑ *ATM gene expression*^15^, ↑ *in TGF-β pathway in* *i-NSCs*^16,47^, ↓ *in cadherin pathway in i-NSCs*^16^, ↑*&*↓ *in ECM functions, cell adhesion and* *cell surface signaling, transmembrane support, and axon guidance in i-NSCs*^16^, ↑*&*↓ *in* *ECM organization, synapse assembly, axon guidance, neurotrophic signaling, and the* *BDNF pathway*^48^, ↑*&*↓ *in developmental pathways and cell adhesion in NPCs*^33^ *and* ↓ *in* *ECM organization, development, and differentiation and neurodevelopmental pathways in* *NPCs*^33^ *and others* ** Functional phenotypes:** • ↑ H3K9 methylation in iPCS^33^ • Proteasome inhibition by MG132 or autophagy inhibition by bafilomycin induces mHTT aggregates i-tissues^14,36^ • Neural rosette formation and neural patterning is delayed^14^ • ↓ neurite length in cortical i-neurons^31^ • ↑ levels of caspase-3/7 activity in i-NPCs^13^ • ↑ number of TUNEL-positive staining iPSCs^15^ i-NSCs^16^ and in differentiated i-neurons^32^ • ↑ P53 and ATM expression and phosphorylation in iPSCs^16^ • ↓ mitochondrial respiration and ATP levels as well as other differences in mitochondrial dynamics i-NSCs^49^
CS77iHD-71nX^38^	71	• ***Gene expression changes:*** ↑*&*↓ *in neurodevelopmental pathways, TGF-β pathway, Wnt* *signaling, DNA damage and apoptosis in iPSCs*^38^
HD70 (GM21756)^34^	70	• ↑ P53 phosphorylation i-tissues?^15,16,34^ • ↑ ATM and P53 protein levels iPSCs^34^ • ↑ γH2AX phosphorylation^34^ in iPSCs.
CS21iHD-60nX^18^	60	• ***Gene expression changes:*** ↑ *in TGF-β pathway in i-neurons*^17^, ↑*&*↓ *in ECM functions* *in i-neurons^17^ and i-NPCs*^17,18^, ↑*&*↓ *in synapse assembly, axon guidance, neurotrophic* *signaling, and BDNF pathway, and key developmental pathways in i-neurons^17^ and* *cortical i-neurons*^31^ ** Functional phenotypes:** • ↑&↓ epigenetic alterations in i-neruons^17^ • ↑ levels of TUNEL-positive staining in iPSCs^18^ • ↑ cell death in i-neruons^18^ • Alterations in neuronal patterning and OCT4 remains high after neuronal induction in i-NPCs^37^ • ↓ intracellular ATP levels in i-NSCs^17^, iPSCs^11^ and i-neurons^18,19,44^ • ↓ bioenergetics and ↓ glycolytic capacity in i-tissues^18,19,44^ • ↑ glutamate sensitivity in i-neurons^18^ • ↑ caspase-3/7 activity in i-neurons^18^ • ↑ P53 phosphorylation in i-tissues^15,16,34^
CS03iHD-53nX^17,43^	53	***Gene expression changes:*** ↑ *in cell cycle and Wnt/β-Catenin signaling in MSN i-neurons*^50^ ** Functional phenotypes:** • Mis-localization of nuclear pore proteins and alterations of the nucleocytoplasmic transport in i-neurons^43^ • ↑ cell death in MSN i-neurons^17^ • ↑ neurite length in MSN i-neurons^17^ • ↑ neurite length in cortical i-neurons^51^ • ↓ population of enduring mitotically active and resistant-to-differentiation subset of proliferating cells amongst MSN i-tissues^50^
iPSHD11, iPSHD22, and iPSHD34^52^	40, 47, and 42, respectively	• ↑ nuclear envelope abnormalities in i-neurons^52^ • ↑ lysosomes and autophagosomes in i-neurons^52^ • ↑ susceptibility to cell death by proteasome inhibition in i-neurons^52^ • ↓ mitochondrial density (only in iPSHD11 iPSHD22)^53^ • ↑ SOCE calcium currents in HD i-neurons^52,54^
CS04iHD-46nX^17^	46	• ***Gene expression changes:*** ↑ *in cell cycle and Wnt/β-Catenin signaling in MSN i-neurons*^50^ ** Functional phenotypes:** • ↑ cell death in i-neurons^17^ • ↑ neurite length in MSN i-neurons^17^ • ↑ population of enduring mitotically active and resistant-to-differentiation subset of proliferating cells amongst MSN i-tissues^50^
HD1 (GM04022) and HD2 (GM02191)^55^	44 and 42 repeats	• ↑ 5-hydroxymethylation in i-NSCs • ↑ DNMT family gene expression in i-NPCs^55^ • ↑ DNA repair gene expression in NSCs and iPSCs^55^
ChiPS31-HD-hiPS^56^	42/44 homozygous	• DNA hypermethylation in iPSCs^57^ • ↑ lysosomes and LC3 BII expression in iPSCs and i-NSCs • ↑ lysosomes and LC3 BII expression^56^

Summary of phenotypes associated with HD i-tissues. ↑ = increased compared with controls, ↓ = decreased compared with controls, ↑&↓ = components or steps of this pathway may be either increased or decreased relative to controls. Gene expression changes are *italicized*. Underlined font indicates phenotypes found across at least two lines with different CAG repeat lengths. * Line may have a slightly different name depending on the study or publication. ** The CAG repeat size listed here is an estimate of the number of contiguous CAG triplets and does not include CAA interruptions, which are likely to occur and have been identified in human^58,59^ and mouse models^60^. *** To generate this list, we focused on the most prominent phenotypes reported between 2012 and 2022. We acknowledge that this list may not be exhaustive, as we may have missed some reports or new phenotypes may have been described since. Note: Not all HD lines that have been generated and studied are listed here. Please refer to the Coriell database (https://www.coriell.org/) for the complete list from the HD iPSC Consortium and for other lists that are publicly available. BDNF, brain-derived neurotrophic factor; ECM, extracellular matrix; HD, Huntington’s disease; i-NPC, neural precursor cell developed from induced pluripotent stem cells; i-NSC, neural stem cell developed from induced pluripotent stem cells; i-neuron, neuron developed from induced pluripotent stem cells; iPSC, induced pluripotent stem cell; i-tissue, tissue developed from induced pluripotent stem cells; mHTT, mutant huntingtin; PPAR, peroxisome proliferator-activated receptor; SOCE, store-operated calcium entry; TGF-β, transforming growth factor beta; TUNEL, terminal deoxynucleotidyl transferase dUTP nick end labeling; VGCC, voltage-gated Ca^2+^ channel.

In 2008, the Daley lab was the first to develop human iPSC-derived HD models and to demonstrate that the fibroblasts of patients with HD could be reprogrammed into pluripotent stem cells^11^. The first HD patient iPSC line harbored 72 CAG repeats^12^ and was subsequently differentiated into GABAergic, DARPP32-positive neurons^13^, demonstrating that iPSCs could be patterned towards striatal neurons, a cell type that is vulnerable to degeneration in HD. Others have also generated iPSCs from patients with HD and differentiated them into neurons that can be patterned into striatal cultures or neural progenitor cells to interrogate HD-related phenotypes^14–19^. The use of HD iPSC models has made it possible to study the links between mHTT, the activity of multiple biological pathways, and pathogenesis^20^. Work in other HD models has found that mHTT disrupts mitochondrial function^21–23^. In addition, neurons depend on proteasome activity^24–26^ and autophagy flux^27^ to remove misfolded proteins like mHTT. Cells that express mHTT show impairment of these clearance pathways leading to mHTT buildup, which accelerates neurodegeneration^28–30^. In the next section, we discuss how the use of human HD iPSC models has aided in our understanding of disease mechanisms, thus providing insights into the development of effective therapies for HD.

**Figure 1.**
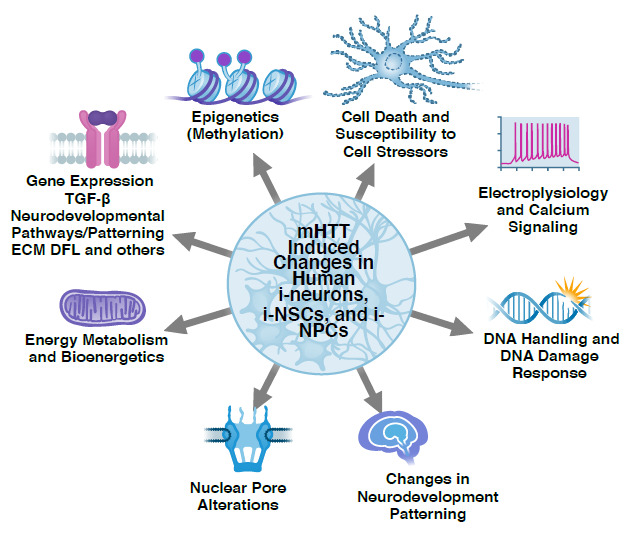
Cartoon summarizing the most prominent phenotypes identified in Huntington’s disease tissues developed from induced pluripotent stem cells. DFL, dermal fibroblast; ECM, extracellular matrix; i-NPC, neural precursor cell developed from induced pluripotent stem cells; i-NSC, neural stem cell developed from induced pluripotent stem cells; i-neuron, neuron developed from induced pluripotent stem cells; TGF-β, transforming growth factor beta.

For simplicity, we refer to neurons, neural stem cells, neural progenitor cells, and tissues developed from iPSCs as i-neurons, i-NSCs, i-NPCs, and i-tissues, respectively. (See Table 1 and Figure 1 for a summary of data on HD iPSC lines.)

## Somatic instability and DNA handling in HD

It is generally believed that symptomatic HD is found in patients with CAG repeat lengths greater than 40. The number of CAG repeats in *HTT* is not permanently fixed and instead undergoes somatic expansion in some human tissues, particularly in the striatum and cortex^61–64^. The cerebellar cortex appears to display the lowest degree of CAG repeat instability^61^, while other brain tissues display a high degree of CAG repeat mosaicism^61,62,65^. Somatic instability in the cortex in HD brains is a predictor of age of onset^63^ and increases with age^66^. Recently, it has been observed that individuals who lack a CAA interruption in the CAG repeat have a significantly earlier age of onset in HD as well as increased somatic instability^58,59,60,67^. Somatic instability is often linked to an altered DNA damage response. Indeed, genome-wide association studies (GWAS) have identified numerous variants significantly associated with DNA damage control pathways that modify HD age of onset, including *FAN1, LIG1*, *MLH1*, *MSH3*, *PMS1*, and *PMS2*^68–73^. Moreover, reducing expression of a subset of these genes in mice caused changes in somatic instability across different tissues, including iPSCs^71,72^. Together, these data suggest that the DNA repair machinery is tightly connected with the somatic instability of the CAG repeats in *HTT* and may play a major role in HD onset and progression.

The number of CAG repeats is generally stable during reprogramming of patient fibroblasts into iPSCs, and, as a general rule, neither iPSC passaging, when some cells in a culture are used to generate a new cell population, nor differentiation into brain cells seems to alter the CAG repeat length significantly^5,18,56^. There are exceptions such as iPSCs from patients with very long CAG repeats (e.g., the HD109 line), where additional expansion of the CAG repeat length can occur with passaging for both iPSCs^45^ and i-NPCs^18^ as well as during differentiation to medium size spiny neurons (MSNs)^45^.

Interestingly, in lines with large CAG expansions such as the HD109 and HD72 lines, knocking down one of the GWAS modifiers of DNA damage control, *FAN1*, exacerbated CAG expansions^45,72^, suggesting that *FAN1* is needed to limit somatic expansion. In fact, perturbations in DNA handling genes and the DNA damage response occur in HD^74,75^. For example, p53 signaling is increased in HD models^35,76^, and HD iPSCs exhibit elevated p53 levels and phosphorylation^15,34^, as well as ATM expression^15^ and phosphorylation^15,34^ and γH2AX phosphorylation^15,34^. All of these proteins are widely used as markers of DNA damage^15,34,77,78^. Surprisingly, p53 expression was found to be decreased in iPSCs and i-NSCs with very long CAG expansions from patients with juvenile-onset HD^39,40^. The reasons for this perplexing finding are currently unclear. In studies employing a number of HD i-neurons with a range of CAG repeat lengths of 66, 71, and 109, Morozko *et al*. (2021)^79^ reported that the SUMO E3 ligase PIAS1 is involved in DNA damage control and that its reduction in HD iPSC neurons normalized HD transcriptional dysregulation associated with DNA damage repair. This served to increase genomic integrity in HD iPSC-derived neurons, supporting a role for SUMOylation in DNA damage repair in HD. Nevertheless, many genes involved in the DNA damage response pathway were found to be altered in HD i-neurons, iPSCs, and i-NSCs compared with controls^15,34,38,55^, further confirming the importance of the DNA damage pathway in the pathology of HD neurons.

## Nuclear pore alterations in HD

The nuclear pore serves as a master regulator for nuclear import and export and thereby controls numerous cellular processes involved in gene transcription and translation^80^. Nuclear pore dynamics may be impaired in neurons vulnerable to HD since the RanGTP gradient, a critical regulator of nuclear pore dynamics, is compromised in HD, and numerous nuclear pore proteins such as NUP62 and RanGAP1 are mislocalized in HD i-neurons compared with controls^43^. Altered nuclear pore functions may be responsible for aberrations of nuclear morphology in HD i-neurons^52^, and alterations in nuclear envelope morphology have been shown in human HD brain tissue as well as i-NSCs^42^. The shape and size of the nucleus are also altered in HD i-neurons^53^, although how this affects nuclear dynamics or function is not understood. As mHTT alters chromatin and gene expression, it is perhaps not surprising that gatekeepers of transport in and out of the nucleus are also disrupted in HD.

## mHTT-induced gene expression changes

Shortly after iPSC technology was introduced^1,2^, the effort to create patient-derived lines was of great interest to the HD field. The National Institutes of Health formed a consortium of HD researchers, called the “HD iPSC Consortium”, to investigate both the transcriptomic changes and functional and structural degeneration associated with HD. This team reported gene expression differences between i-NSCs derived from controls and individuals with juvenile- and adult-onset HD, harboring 180 and 60 CAG repeats, respectively. Their microarray studies identified differential expression of genes in numerous pathways involved in development, cell cycle, proliferation, and nervous system function, and they found a subset of gene expression changes that were unique to the juvenile onset cells(CAG180 line)^18^. Later, the HD iPSC Consortium performed RNA sequencing (RNA-seq) analysis on a subset of the same lines (HD i-neurons harboring 60 and 109 CAG expansions with multiple clones of each) and again found a dysregulation of key developmental pathways and master regulators of neurogenesis^17^. More than half of the genes altered were involved in nervous system function, cellular and tissue development, and axon guidance^17^.

One of the mechanisms by which mHTT is suspected to cause neurodegeneration is by altering gene transcription. This is supported by the findings of the Ellerby group^16^, who used i-NSCs generated from HD iPSCs that harbored 72 CAG repeats and compared their gene expression profiles with gene-corrected control lines^11^ by using microarray analysis. They found that the transforming growth factor beta (TGF-β) signaling pathway was significantly upregulated as a consequence of CAG repeat expansion^16^. The TGF-β signaling pathway supports numerous cellular activities involved in development and growth, including proliferation, differentiation, patterning^81^, apoptosis, and tumor suppression^82^. This finding has been corroborated: dysregulation of the TGF-β pathway has been observed in i-NSCs harboring 72 CAG repeats^16,47,48^, 180 CAG repeats^32^, and 60 and 109 CAG repeats^17^. In addition, SMAD transcription factors, the downstream effectors of TGF-β signaling, have been implicated as central players in the regulation of HTT itself^41^.

Another major cellular pathway dysregulated in HD i-tissues is the extracellular matrix (ECM). The ECM provides structural support to cells and modulates the signaling events and force generation required for cell communication, migration, polarity, and movement^83,84^. Genes related to ECM functions such as cell adhesion and cell surface signaling, transmembrane support, and axon guidance are altered in HD i-NSCs and i-neurons^16–18^. In particular, cadherins, calcium-dependent molecules that regulate cell adhesion and connection^85^, were among the most prominent downregulated genes in HD i-NSCs with 72 CAG repeats^16^.

RNA-seq of i-neurons derived from this same HD line^48^ and its gene-corrected counterpart as well as from other HD lines harboring 60 and 109 CAG repeats^17^ revealed more complex gene dysregulation in numerous pathways, such as synapse assembly, axon guidance, neurotrophic signaling, and the brain-derived neurotrophic factor (BDNF) pathway^17,48^. RNA-seq on cortical-patterned i-neurons harboring 77 and 109 CAG repeats compared with controls showed similar patterns of transcriptional dysregulation in nervous system development, ECM, and axon guidance molecules^31^. The changes in synaptic assembly and axon guidance may underlie the synapse degeneration and dysfunction of neuronal circuitry, while loss of supportive neurotropic signaling may contribute to neuronal death over time.

Many of the genes downregulated in HD i-neurons are transcriptional regulators related to neuronal development. This includes REST, which represses genes involved in neuronal fate in non-neuronal cells^86^ and prevents neuronal differentiation in stem cells^87^, and NEUROD1^17^, a key regulator of neuronal differentiation. Of interest, activation of NEUROD1 by either its overexpression or stimulation by a small molecule has been shown to ameliorate disease phenotypes in HD neurons. Other studies have reported similar gene expression changes in developmental pathways^31,32,38^. Together, these findings suggest that cellular activities involved in neuronal maturation are dysregulated in HD, which is consistent with other developmental phenotypes observed in HD i-tissues.

## Epigenetic changes in HD

One of the mechanisms controlling gene expression involves epigenetic modulators, and epigenetic changes underlie many of the transcriptional alterations in HD^88–90^. Studies have shown altered epigenetics in HD iPSCs, including changes in histone methylation and acetylation. Transcriptional changes identified by RNA-seq were highly correlated with alterations in the H3K4me3 and H3K27ac epigenetic marks^17^. In i-NSCs derived from HD patients harboring 71 CAG repeats, H3K9 methylation was increased compared with controls, and this change was associated with altered expression of genes involved in proliferation, development, differentiation, ECM, and axon guidance^33^. Changes in histone H3K9 methylation have also been observed in postmortem HD brains^91^.

ATF7IP, which regulates H3K9 methylation, interacts with wild-type HTT, but this interaction is lost in iPSCs harboring 71 CAG repeats^33^. mHTT directly alters the deposition of H3K27me3 at active sites of mouse embryonic stem cells (ESCs) and neural precursor cells (NPCs), suggesting that mHTT may directly regulate histone methylation^92^. Sequence motif analysis revealed that the largest changes in H3K27ac marks occur near transcription factor binding sites at genes involved in neurodevelopmental pathways^17^.

Another study examining a rare homozygous CAG expanded line harboring 42/44 CAG repeats reported DNA hypermethylation in promoter regions, and the extent of hypermethylation increased as the cells matured and patterned^57^. Methylation was observed at the promoter region of WDR5, a chromatin remodeler that itself regulates methylation of genes involved in pluripotency^57^. Finally, there was a global increase of 5-hydroxymethylation in HD i-NSCs that harbor 43 and 44 CAG repeats compared with controls^55^. These data suggest that CAG expansion in *HTT* or the resulting glutamine expansion in mHTT (or both) may alter the fate of neuronal development by exerting effects at the epigenome level.

## Inclusion bodies and proteostasis

An important use of HD i-neurons is that they allow for the study of the role of endogenous levels of mHTT on cell health rather than requiring overexpression of the protein, as in most other cell models of the disease. mHTT is misfolded and forms inclusion bodies (IBs) in cells of the HD brain. Pathological mHTT inclusions have long been associated with HD and are found in the brains of murine models of HD, as shown primarily by immunohistochemical staining using antibodies against mHTT, such as EM48 or MW8^93–97^. Some investigators have reported that HD i-tissues in culture do not develop EM48- or MW8-positive aggregates or intranuclear inclusions^14,18^. However, HD i-NSCs grafted into murine brains at postnatal day 2 exhibited EM48-positive aggregates after about 33 weeks^14^, suggesting that aging and the brain microenvironment may be required for pathological mHTT IB formation. Nekrasov *et al*.^52^ also reported the presence of EM48-positive inclusions in 6-month-old i-neuron cultures. However, no quantification was performed, so the extent of aggregation in this context is unclear^52^.

The increased levels of mHTT, and its aggregation, are due in part to impairment of the proteostasis system^30,98,99^. Proteostasis entails the breakdown of misfolded proteins. The breakdown of misfolded proteins in cells normally occurs by two main pathways: the ubiquitin-proteasome system and autophagy. HD iPSCs and i-NSCs with moderate-length CAG repeat expansions (42–45 CAG repeats) display increased lysotracker staining, indicating altered lysosome activity compared with controls^56^. Another study employing cells with moderate-length CAG repeat expansions found increased lysosomes and autophagosomes in HD i-neurons as well as increased mitophagy^52^. Likewise, early-stage HD i-NSCs exhibit increased markers of autophagy compared with control i-NSCs^56^. These findings are consistent with observations in HD mouse models^100,101^ and suggest that proteostasis is activated in these pluripotent cells, possibly reflecting the cells’ attempt to clear toxic mHTT.

However, the consequences of this activation are not understood. Numerous studies report that mHTT causes significant dysfunction of the proteostasis system, leading to neurodegeneration^98,102^. Treatment with the autophagy inhibitor 3-MA or the proteasome inhibitor MG132 increases cell death in HD i-neurons^18,52^, confirming that HD i-tissues may be more sensitive to perturbations of protein homeostasis.

Indeed, treatment with the proteasome inhibitor MG132 or the autophagy inhibitor bafilomycin increases the accumulation of mHTT aggregates in HD iPSCs harboring 71 and 180 CAG repeats^14,36^. Similarly, knocking down one of the most highly expressed E3 ubiquitin ligases, UBR5, in iPSCs results in the generation of mHTT aggregates and increased levels of mHTT^36^. Oxidative stress induced by menadione also results in EM48-positive aggregates in HD i-neurons harboring 99 CAG repeats^103^. Furthermore, mHTT may stress proteasomal pathways, as it has been shown that HD i-NSCs harboring 46, 70, and 99 CAG repeats have an increase in ubiquitinated proteins and delayed degradation of proteasome substrates^103^.

To study the mechanisms by which mHTT may impact proteostasis, our group has used a longitudinal single-cell imaging technology, robotic microscopy (RM)^104,105^, that can observe i-neurons from HD patients over long periods of time. The high sensitivity of RM allows it to detect disease phenotypes in HD i-neurons without the need for cellular stressors. Using RM, we found that i-neurons from patients with HD harboring 46, 53, 60, or 180 CAGs displayed signs of degeneration such as the retraction of neurites and loss of soma, resulting in a higher cumulative risk of death than controls^17,18^.

RM analyses also showed that the formation of mHTT IBs in neurons does not cause degeneration but rather serves as a coping mechanism to slow neurodegeneration^104,105^. To directly study the impact of mHTT on mechanisms of protein clearance, an optical pulse labeling (OPL) technology using photoswitchable probes was developed. OPL allows us to monitor turnover of mHTT and autophagic flux or proteasome activity in single cells^30,106^. Using OPL and employing Bayesian regression modeling, we found that the mean lifetime of mHTT in single neurons was a greater predictor of neurodegeneration than absolute levels of mHTT^30^. Autophagy was determined as the prime cellular mechanism of clearance of mHTT, and small-molecule drugs that increase autophagy increased clearance of mHTT and reduced mHTT toxicity and the risk of death in a primary neuron HD model^106^, supporting the potential utility of small-molecule autophagy inducers to slow the progression of HD.

RM and OPL analyses were used to discover that deubiquitinase Usp12 protects against mHTT-induced neurotoxicity in a primary rodent model of HD and in HD i-neurons from a patient with 109 CAG repeats^107^. Interestingly, the catalytic activity of Usp12 was not needed for neuroprotection. Usp12 was found to protect against mHTT toxicity by inducing autophagic flux in neurons, and the enzyme was proposed to be an important regulator of neuronal proteostasis in HD^107^.

## Neuronal development

HD i-neurons show a number of aberrations in neuronal patterning^17,32,37,108^ as well as a persistence of mitotic populations^50^. Global changes in neurodevelopmental gene expression have been described in HD and are likely linked to the changes in neuronal differentiation and connectivity.

Nestin is an intermediate filament protein and has been widely used as a marker of NSCs^109^. Genetic deletion of nestin reduces neuronal self-renewal and increases apoptosis^110^, whereas overexpression increases proliferation and results in larger brain and heart size^111^. In HD iPSC lines harboring 60, 109, and 180 CAG repeats, there is an increased number of nestin-positive cells as well as overall increased nestin expression compared with controls^108,112^. The expression of numerous cell cycle-related genes dramatically increases in HD iPSC lines harboring 46 and 53 CAG repeats, which likely explains the observation of a population of mitotically active cells that are resistant to differentiation^50^. These changes appear to be directly related to increased Wnt/*β*-Ca signaling as they are rescued by inhibition of this pathway^50^.

The developmental impairment in HD i-tissues can occur early in differentiation, even before neural patterning begins. Upon exposure to SMAD inhibition, a typical neural induction methodology^113^, the pluripotency marker OCT4 is normally downregulated^114^. In iPSCs that harbor large CAG repeat expansions of *HTT*, the downregulation of OCT4 and conversion to neural markers such as PAX6 are significantly delayed^37^. Neural rosette formation, a step in i-NSC differentiation, is also deficient in HD-iNSCs^14,32,37^. Gene correction of the CAG expansion normalizes rosette formation^32^. The delay in development appears to persist well into later stages, as striatal markers, such as CTIP2 and DARPP32, and pan neuronal markers MAP-2 and β III-tubulin are reduced in HD i-neurons compared with controls^37^. These developmental aberrations were restored when HTT expression was reduced by a synthetic zinc finger protein repressor^37^. When HD iPSCs were patterned to a cortical fate, the neurons harboring 77 and 109 CAG repeats exhibited gene expression and electrophysiological phenotypes that were consistent with delayed maturation^31^.

To gain insight into how mHTT alters neuronal morphology, several groups have examined neurite length in HD i-neurons because neurite length may be an indicator of synapse formation as well as synapse pruning. In i-neurons patterned towards an MSN fate, neurite length is increased in HD lines harboring 46, 53, or 109 CAG repeats, consistent with previous reports of ESCs that harbor CAG expansions and observations in the brains of patients with HD^115–117^ and with the finding that numerous axon guidance molecules are altered in HD^17^. Another study reported that when patterned towards cortical neurons, i-neurons with 53^51^, 77, 109, or 180 CAG repeats had significantly shorter neurites than the controls did^31,51^. Notably, in this later study, measurements were made much later in differentiation; thus, morphological changes may vary depending on the stage of differentiation and the cell type.

## Altered bioenergetics

In mouse models of HD, CAG repeat expansions are associated with mitochondrial pathologies such as altered respiration, energy generation, and metabolic rate^118–121^. Similar findings have been observed in HD i-tissues. For example, HD i-NSCs with various CAG expansions exhibit decreased ATP levels compared with controls^18,19,44,49^, suggesting impaired energy production. HD i-NSCs and HD iPSCs from lines harboring 72 CAG repeats display numerous changes associated with mitochondrial dysfunction, biogenesis, dynamics, and morphology, and many of these phenotypes were restored after removal of the CAG repeat^49^. A quantitative proteomics analysis performed on i-neurons harboring 60 and 109 CAG repeats revealed a unique set of differentially expressed proteins in metabolic pathways and bioenergetic processes that could not be explained by gene expression changes^17,19^. This suggested that the metabolic defects may be due to post-translational events rather than transcriptional dysregulation. HD i-NSCs have also been shown to have compromised glycolysis^19^, and HD i-neurons harboring 42–44 repeats have lower mitochondrial density than controls do^53^. However, in an exhaustive examination of numerous aspects of mitochondrial function, such as mitochondrial membrane potential, ATP levels, respiration, and oxygen consumption rates, Hamilton *et al*. observed no differences between control and HD i-neurons containing 46, 53, or 72 CAG repeats^122^.

The reason for the differences in these studies is unclear. They may reflect differences in differentiation methods. Hamilton *et al*. (2020)^122^, who observed no apparent bioenergetic changes in HD i-neurons, used a method that generated embryoid bodies before generating neural rosettes, which were then harvested by rosette selection medium before differentiation. By contrast, most of the studies that showed changes in mitochondrial function used different methods for the neuronal differentiation, which may have led to cultures with a very different cellular composition. It may also be that the harvested i-NSCs are sub-stratified to generate a slightly different neuronal population of MSNs. Differentiation of MSN cultures is primarily validated by the staining of the MSN marker, DARPP32. However, in our experience (unpublished results), existing antibodies against DARPP32 are problematic and have exhibited variations in sensitivity and specificity, thereby rendering them unreliable in characterizing the outcomes of a particular differentiation. In other words, without better markers for the different differentiation outcomes, it remains difficult to compare findings from different groups and to ascertain whether and at what stages bioenergetics may differ between HD and control neuron populations differentiated *in vitro*.

## Mechanisms of cell death in HD

Patients with HD exhibit a gradual degeneration of striatal MSNs that contributes to movement abnormalities^123–126^. Cerebral cortical cells also degenerate over time and likely contribute to cognitive deficits as well as motor abnormalities in HD^124,127^. Numerous cellular signals may contribute to cell death in HD, including altered calcium signaling^128^, loss of the supportive trophic factor BDNF^129,130^, proteolytic cleavage of mHTT^131–133^, and activation of the cell death pathways such as apoptosis and necrosis^134^.

HD i-neurons and i-tissues have been used to further refine our understanding of the mechanisms involved in neurodegeneration and cell death in HD. For example, apoptosis is known to activate caspases, which initiate a cascade of downstream signaling events leading to cell death^135^. Several studies have found activation of caspases in HD i-tissues. The Ellerby group has shown that, after growth factor withdrawal, HD i-NSCs have increased levels of caspase-3/7 activity^13^, and these findings have been confirmed by other groups and in different lines^18,136^. Gene correction of the 72 CAG expansion prevented the increase in caspase-3/7 activity, suggesting that activation of the caspase-mediated pathway is related to CAG expansion in *HTT*^16^. However, in an independent study of an HD iPSC line with just 42–44 CAG repeats, no elevation of caspase-3 activity was observed, suggesting that activation of this enzyme may be dependent on the length of the CAG repeat^56^.

Growth factors such as BDNF are critical for the survival of brain neurons, and levels of BDNF are diminished in HD brains. The reduction in BDNF is due to both decreased expression^129,130,137,138^ and altered BDNF transport in cortical neurons, which reduces BDNF release in the corticostriatal synapse^139,140^. HD i-tissues have increased sensitivity to loss of trophic factors, and after growth factor withdrawal, increased numbers of terminal deoxynucleotidyl transferase dUTP nick end labeling (TUNEL)-positive cells have been observed in HD i-NSCs^16^ and in differentiated HD i-neurons^32^. Specifically, reducing the level of BDNF in the media was shown to increase the number of TUNEL-positive HD i-neurons^18,35,44,108^ as well as the cell death rate and caspase-3/7 activity^18^. Overexpression of BDNF prevented the increased cell death rate in HD i-neurons^18^, as did treatment with an agonist of TrkB, a receptor that mediates the actions of BDNF, suggesting that impaired TrkB signaling due to loss of BDNF makes HD i-neurons susceptible to cell death^108^. Interestingly, the population of cells most susceptible to BDNF withdrawal appears to be nestin-positive progenitors^108^, suggesting that underdeveloped neurons are more sensitive to the insults of mHTT than mature cells.

The primary excitatory neurotransmitter in the brain is glutamate, and excess glutamatergic transmission is known to cause neurodegeneration in HD. Like HD brain tissues, HD i-tissues are more sensitive to glutamate, which induces calcium dysregulation and increased numbers of TUNEL-positive cells^18^. Furthermore, mHTT increases glutamate release from i-neurons, which in turn hyperactivates the glutamate *N*-methyl-d-aspartate receptors (NMDARs)^141^, resulting in excess calcium signaling. It also leads to downstream changes such as recruitment of extrasynaptic NMDARs, decreased CREB-mediated transcription, and induction of apoptosis^128^. Aberrant calcium signaling through store-operated calcium entry (SOCE) has also been observed in HD i-neurons, as inward calcium currents are about two-fold higher in HD i-neurons (notably in numerous patient lines that came from typical and juvenile-onset HD and contained 40–47 and 76 CAG repeats, respectively) compared with controls^46,52,54^. Calcium dysregulation in HD has long been studied, and aberrant SOCE signaling is likely a major player in this observation^142^.

CAG expanded lines are also uniquely sensitive to cellular stressors, particularly to oxidative stressors such as H_2_O_2_^18,143^. In HD i-neurons, H_2_O_2_ was also shown to increase expression of the DNA damage marker γH2AX^143^. Exposure to the endoplasmic reticulum stress inducers thapsigargin and dithiothreitol increased nuclear indentations in HD i-neurons, with 42 and 44 CAG repeats significantly more than in control i-neurons. However, the molecular nature of this pathology is unclear^53^. Studies by Machiela *et al*.^144^ reported a role for aging in accelerating the sensitivity of HD i-neurons to cellular stressors. In a study of HD iPSCs differentiated towards the astrocytic lineage, both cells with the juvenile-onset 109 CAG repeats and the adult-onset 50 CAG repeats exhibited cytoplasmic vacuoles not present in control cells. These vacuoles were exacerbated by the cellular stressor chloroquine^145^. This is a curious observation as cytoplasmic vacuolization is thought to occur after cytotoxic stimuli such as bacterial pathogens or exposure to toxic compounds and precedes activation of cell death pathways^146^.

## Isogenic embryonic stem cell lines to study CAG expansions

When iPSCs were first used to study HD, it remained controversial whether there were major differences compared with ESCs and whether they were better or worse for disease modeling^147^. It has been reported that they are functionally similar in terms of gene expression^148^ and differentiation potential^149,150^. Human ESCs have been used in numerous studies to study the effect of CAG expansions. Unlike iPSCs derived from patients, ESCs are unlikely to carry a CAG expansion unless derived from patients with HD. Normal ESCs have been engineered to develop allelic series of isogenic lines with varying numbers of CAG repeats^151,152^, which is a powerful way to avoid differences in genetic backgrounds when comparing different lines, as described in the next section. One allelic series was generated in the RUES2 line^153^ using clustered regularly interspaced short palindromic repeats (CRISPR) technology and includes lines with 45, 50, 56–58, 67, and 72–74 CAG repeats^151^. These lines displayed developmental abnormalities^154^, such as yielding an increased number of progenitor cells^154^ and forming aberrant rosettes^151,154^. Other novel phenotypes such as disruptions in cytokinesis and chromosomal instability^151^ were detected, and changes in gene expression in developmental pathways previously detected in iPSCs were also found^151,154^.

Another isogenic CAG allelic series, coined IsoHD lines^152^, was developed in the long-established H9 ESC background^151,155^. The CAG repeats were engineered using transcription activator-like effector nuclease (TALEN) technology, and the series consists of lines with 30, 45, 65, and 81 CAG repeats. The authors investigated numerous phenotypes; notably, many of them were sensitive to CAG repeat length, including defects in mitochondrial respiration, increased reactive oxygen species, and increased sensitivity to DNA damage^152^. Furthermore, the authors took an especially powerful approach to elucidate cell-specific phenotypes by generating different lineages and captured both cell-specific and CAG repeat-specific changes in gene expression at both the RNA and proteomic level^152^. Another study differentiated a subset of these lines into microglia and also found increased activation of reactive oxygen species as well as susceptibility to stressors and increased cytokine production in the cells with CAG expansions compared with isogenic controls^156^. Such allelic series of CAG lengths are powerful tools to dissect the contribution of the CAG expansion without the confounding effects of other genomic or epigenomic differences compared with control lines.

## Transdifferentiation of HD fibroblasts directly to neurons

Recently, researchers have investigated the potential of directly converting fibroblasts into specific cell types rather than passing through a pluripotent cell stage^157,158^. This inventive method is believed to retain many of the epigenetic marks embedded within the chromatin, including marks associated with aging, which may be especially important in the context of neurodegenerative disease modeling, as disease onset is typically late in life^159^. In one study^157^, the authors expressed the brain-enriched microRNAs miR-9/9* and miR-124 in HD patient fibroblasts and induced expression of MSN markers. Most importantly, these MSN-like cells displayed aggregates of mHTT and IB formation, which is remarkable given that the parental fibroblasts showed no signs of mHTT aggregation^157^. In addition, the transdifferentiated HD cells exhibited phenotypes associated with neurodegeneration, such as increased oxidative damage, aberrant mitochondrial function, and increased cell death, and these differences correlated with the disease stage of the patients^157^. Because these cells harbor numerous neurodegenerative marks associated with HD, this approach has great potential. However, there still are several caveats to their use. First, the source material is limited. Unlike iPSCs, fibroblasts tolerate only a finite number of passages. Another is that each experiment requires a fresh batch of fibroblasts, which again becomes limiting. Transdifferentiation is long, complex, and difficult, so batch issues become challenging, which reduces the sensitivity, rigor, and reproducibility of the system. The last caveat is that one cannot make gene-corrected controls, which can confound the interpretation of phenotypes found associated with CAG expanded lines.

## The pros and cons of using iPSC technology to study HD

Most of this review has focused on emphasizing the contributions of human iPSC technology to the study of HD and the advantages of this approach. An additional advantage is that iPSCs provide a relatively unlimited source of HD i-neurons that can be employed in high-throughput screens to identify potential therapeutics to treat the disease. Because of the availability of a large number of HD lines from patients with varying numbers of CAG repeats^12,15,17,18,31,34,38,52,55,56^, one can test potential therapeutics for efficacy in a range of genetic backgrounds and disease severity^160^. This is important because for most neurodegenerative diseases, the models employed, including human iPSC lines, are from patients with rare, inherited forms of the diseases that may not be reflective of the general patient population, where disease etiology is unknown. This is not the case in HD, which is unquestionably caused by the expansion of CAG repeats in the *HTT* gene. Nevertheless, it has become clear that variants in genes other than *HTT* contribute to HD^69^. Using the human HD iPSC lines, it is possible to understand the role of these other variants in the disease since these variants may not be expressed or may not affect disease in the same way in animal models.

That said, there are limitations to the iPSC technology. iPSC lines can come from individuals with diverse genetic backgrounds, and this heterogeneity can confound the interpretation of findings^161–165^. This problem has been addressed with great success thanks to the use of gene-editing strategies such as CRISPR^166^ or TALENs^167^. These technologies allow researchers to either correct or introduce mutations of interest in a given line or genetic background (or both)^168–170^. Using gene correction to make isogenic control lines is a powerful strategy to confirm that disease-related phenotypes are due to the mutation of interest and not to clonal variation, cellular heterogeneity, or another mutation or variant in the genetic background. However, generating gene-corrected lines may not entirely solve these problems as genetic background can still influence cell phenotypes^165^. Nevertheless, CAG expanded lines have proven to display robust cellular changes compared with their respective isogenic control lines^13,16,32,49^. Additional heterogeneity and confounding factors come from differences in methodologies between experiments and labs, which can produce different subsets of cell types and complicate comparisons between lines^6,171^. Methods of cell differentiation can be inefficient and complex and result in cultures with heterogeneous cell populations. The longer and more complex the protocol, the more opportunity for batch variation from experiment to experiment, which can confound interpretation of phenotypes. Using linear mixed models or more sophisticated statistical measures to account for all of the batch variation is much needed in the field.

Another challenge with iPSCs is that the reprogramming process changes the epigenetic marks, many of which may influence disease-related phenotypes^172^. While studies have been performed to investigate epigenetic differences in HD using iPSC lines, the process of generation of iPSC lines can vary^173–176^, which can confound the interpretation of the effect of patient-specific epigenetic marks. While epigenetic studies can be done directly on the patient fibroblasts, the use of these cells provides limited insight into the role of epigenetics in creating vulnerability to selective neuronal populations in HD. The use of transdifferentiation technology, as described in the previous section, to study HD^157^ may overcome these limitations as neurons can be generated directly. However, transdifferentiation is still in its early stages, and as fibroblasts have only limited self-renewal potential in culture, transdifferentiation from fibroblasts may never generate the number of cells necessary for high-throughput assays.

## Critical view of the topic

Most of the iPSC models of HD are derived from patient cells, and as such, they harbor the actual human disease mutations and genetic background contributing to disease. While most of our discussion so far has emphasized MSNs as the main target of HD, iPSCs can be differentiated into other types of i-neurons as well as into other cell types, including astrocytes and microglia, the immune cells of the brain. Scientists can therefore use human iPSC models to reproduce some of the cell-cell dependencies that underlie neuronal dysfunction, degeneration, and death and to investigate the role of inflammation in neurodegeneration. This can provide insights into potential molecular targets in humans that might be useful for developing disease-modifying therapeutics. Furthermore, it is possible to generate the human i-neurons most vulnerable to HD (MSNs and cortical neurons)^177^ from large numbers of different patients with HD to identify common disease mechanisms. Since not all aspects of pathogenesis are dependent on CAG repeat length, the *in vitro* human cell models also provide an approach to screen for potential genetic modifiers that may impact disease onset, progression, and symptomology, which also could be molecular targets for drug discovery.

Some have suggested that findings from the human HD i-neurons and i-tissues may be more reflective of disease processes in patients than animal models since these cell populations express the same genetic mutations and, in the case of transdifferentiated cells, may also maintain the epigenetic status of the patients. However, we can learn only so much from *in vitro* studies, and findings from *in vitro* systems may or may not correlate with the disease pathology and behaviors of patients with HD. Both types of models may be necessary to better understand the pathogenesis of HD and to develop novel approaches for disease treatment.

## Future perspectives

New artificial intelligence technology and especially deep learning (DL) methods are providing unique ways to use imaging to make predictions on cell fate without invasive procedures of biosensors^178,179^. This is important because neurodegeneration is heterogeneous in HD, as it is in all neurodegenerative diseases. This heterogeneity is visible at the level of cell models as well, as cultures of human i-neurons carrying the same HD mutation can harbor cells that resist degeneration adjacent to cells that die rapidly. DL technology, with its ability to record and analyze large numbers of individual cells and correlate cell biology to cell fate, can help us unravel the basis of this heterogeneity.

Furthermore, the development of HD i-neurons grown in three-dimensional organoids can provide new approaches to study the degeneration of HD i-neurons under conditions that better maintain their cytoarchitecture than two-dimensional monolayers can. These technologies can also begin to provide the basis for studying dysfunction of neuronal circuitry across distances in the brain, such as cortical-striatal-thalamic circuits, that may contribute to the degeneration of specific cell populations in the brain.
